# Tomotherapy as a tool in image-guided radiation therapy (IGRT): theoretical and technological aspects

**DOI:** 10.2349/biij.3.1.e16

**Published:** 2007-01-01

**Authors:** S Yartsev, T Kron, J Van Dyk

**Affiliations:** 1 Physics and Engineering, London Regional Cancer Program, London Health Sciences Centre, London, Ontario, Canada; 2 Department of Physical Sciences, Peter MacCallum Cancer Centre, East Melbourne, Australia; 3 Departments of Oncology and Medical Biophysics, University of Western Ontario, London, Ontario, Canada

**Keywords:** Image guidance, helical tomotherapy, radiation therapy, IGRT

## Abstract

Helical tomotherapy (HT) is a novel treatment approach that combines Intensity-Modulate Radiation Therapy (IMRT) delivery with in-built image guidance using megavoltage (MV) CT scanning. The technique utilises a 6 MV linear accelerator mounted on a CT type ring gantry. The beam is collimated to a fan beam, which is intensity modulated using a binary multileaf collimator (MLC). As the patient advances slowly through the ring gantry, the linac rotates around the patient with a leaf-opening pattern optimised to deliver a highly conformal dose distribution to the target in the helical beam trajectory. The unit also allows the acquisition of MVCT images using the same radiation source detuned to reduce its effective energy to 3.5 MV, making the dose required for imaging less than 3 cGy. This paper discusses the major features of HT and describes the advantages and disadvantages of this approach in the context of the commercial Hi-ART system.

## INTRODUCTION

Imaging has always been a necessary prerequisite for radiation therapy. Presently, an intense interaction between these two fields of technology is observed. The discovery of X-rays more than a century ago provided the possibility to locate internal organs in the human body and plan radiation delivery with rectangular fields using two-dimensional (2D) transmission images up to the mid-1970s.

The introduction of computed tomography (CT) in clinical practice resulted in high quality 3D images, which allowed precise definition of tumour shape and location. This information motivated technology development, which would allow planning and delivery of radiation in a more conformal way aiming to give enough dose for disease elimination while sparing healthy tissues.

Technological advances in radiation oncology such as three-dimensional conformal radiation therapy (3DCRT) and intensity-modulated radiation therapy (IMRT) allow the shaping of the dose distributions in patients, with a very high degree of conformity and precision [1]. The application of high-dose gradients provides opportunities for escalating tumour doses resulting in a better chance of the elimination of cancerous cells while still sparing healthy, sensitive organs. At the same time, such highly localised dose distributions may result in a partial target miss and/or risk of organ damage if on the day of treatment the patient setup and/or anatomy are different from that of the imaging study used during planning. If changes in the patient’s anatomy are not detected, the treatment could be compromised [2].

Several solutions to correct the position of the target immediately before (or during) treatment have been developed and clinically implemented including fiducial marker implants [3-6], optical positional guidance [7,8], MRI [9], ultrasound [6,10-18], and daily CT imaging [10,18-26]. Each of these techniques has some positive (better targeting, smaller margins) and negative (increased labor and cost, longer treatment times) features and their detailed clinical assessments with respect to specific disease sites are underway.

In the current literature, the term ‘image-guided radiation therapy’ (IGRT) or IG-IMRT is employed to refer to newly emerging radiation planning, patient setup and delivery procedures that integrate image-based tumour definition methods, patient positioning devices and/or radiation delivery guidance tools [27]. IGRT is a necessary companion of improved treatment planning and better radiation delivery.

Helical tomotherapy (HT) is a novel radiotherapy concept that combines elements from a helical CT scanner with a megavoltage (MV) linear accelerator [28-30]. The idea to include a MV imaging system for setup and dose verification was already put forward in 1993 in the first publication on helical tomotherapy [31]. In the initial version of IGRT with on-board MVCT implemented in the commercially available Hi-ART model, MVCT allows daily patient setup verification and repositioning. In the future, MVCT will also be used for imaging patients followed by quick planning for rapid treatment of emergency cases [32] and for real-time image collection during treatment delivery [20]. In this report, the basic principles of imaging with tomotherapy are discussed. In the companion article, we review the first results of HT use in clinical practice.

## THE HELICAL TOMOTHERAPY APPROACH TO IGRT

**Figure 1:**
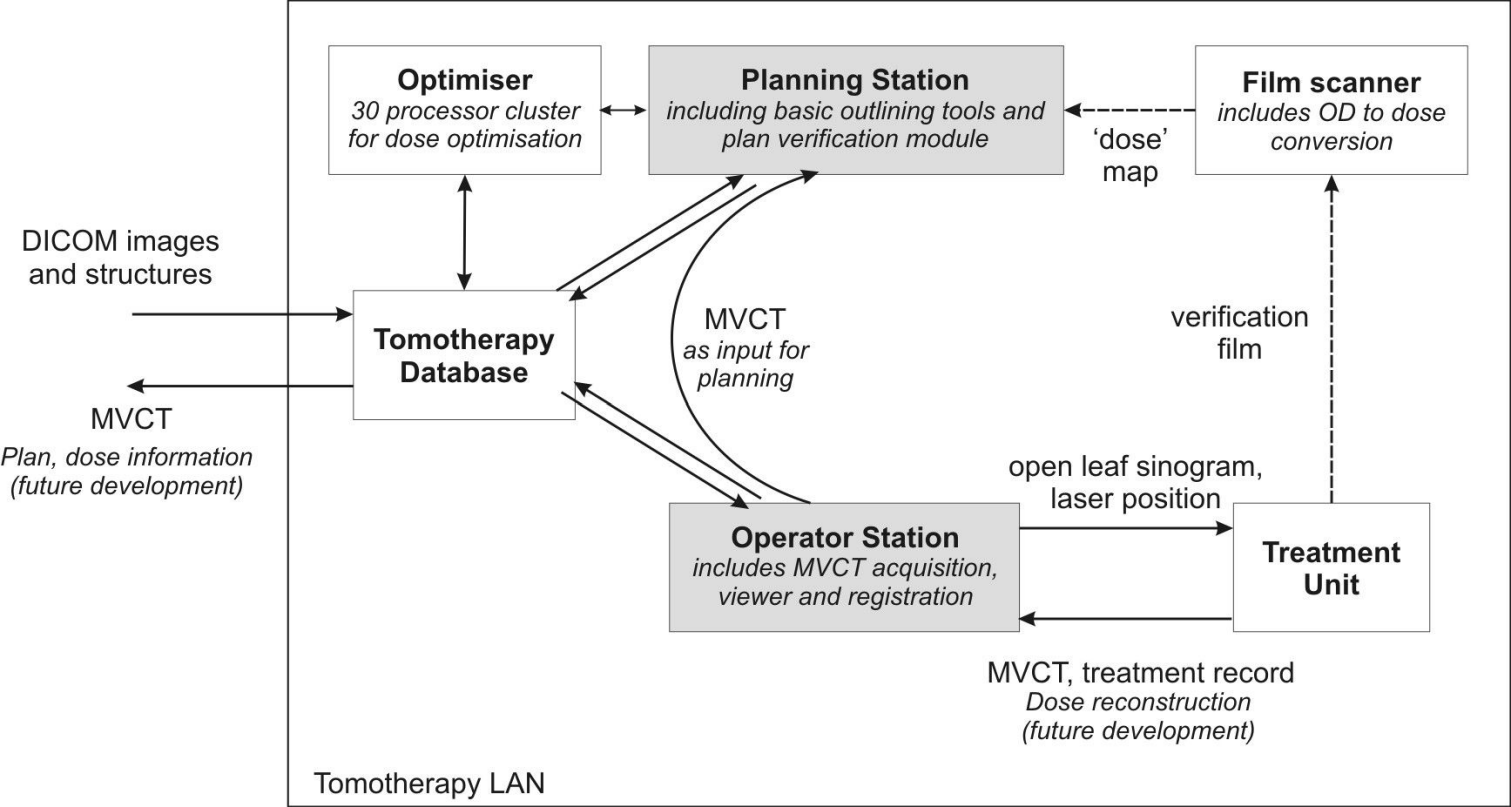
The schematic components of the tomotherapy unit.

The major components of the helical tomotherapy system are shown schematically in Figure 1. The patient is scanned on a diagnostic kilovoltage CT (kVCT) unit prior to HT planning and all structures (gross tumour volume, planning target volume and every sensitive organ that needs to be protected) should be outlined. Patient CT data and structure set are transferred to the HT database using DICOM protocol. This information will be used for inverse planning on the planning station and also as a reference for image guidance on the operator station where the planning kVCT image is compared to the MVCT image taken immediately before treatment. Creation of digitally reconstructed radiographs is not necessary as planning kVCT images will be directly compared to MVCT verification images.

### Radiation delivery

**Figure 2:**
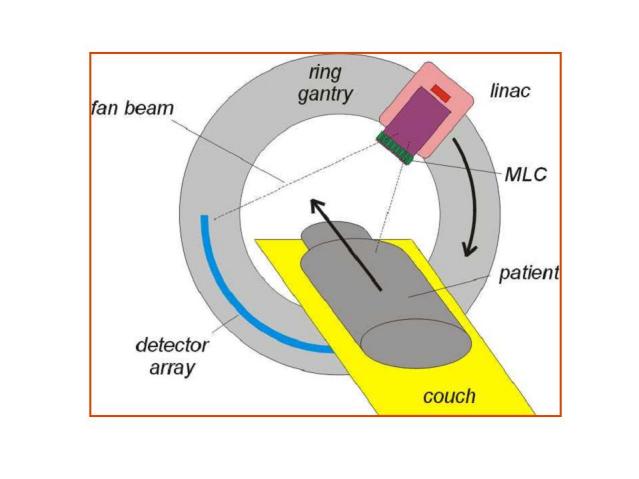
Schematic drawing of a helical tomotherapy unit.

On the HT unit, a conventional 6 MV linear accelerator and a detector array system are mounted opposite each other on a ring gantry that continuously rotates during the imaging and treatment procedures while the couch translates at a constant speed through the gantry as schematically shown in Figure 2. The design ensures minimal gantry sag and, provided the unit is properly aligned, the centre of rotation for radiation and mechanical components should be within 1 mm [33]. No flattening filter is used and the X-ray beam with an output of about 10 Gy/min at isocentre is collimated to fan beam geometry with a width of 40 cm and a fan beam thickness (FBT) variable from a few millimeters to 50 mm. Orthogonal to the fan beam width is a binary (i.e. ‘either open or shut‘) multi-leaf collimator (MLC). Its 64 leaves are divergent with the beam and project to 6.25 mm width at isocentre. The transit time for the leaves is between 20 and 30 ms for the largest fan beam thickness. As the unit is specifically designed for IMRT, the leaf thickness (10 cm tungsten) is thicker than in most conventional MLCs and the overall shielding of the head is better. Therefore, leakage radiation to the patient is generally low despite being treated with long beam at times. Jeraj *et al *found the out-of-field leakage to be less than 0.1% [34], which would result in 1% dose to the periphery of the patient even in long and complex treatments [35].

For planning and dose delivery, the full gantry rotation is divided into 51 projections. Each projection is characterised by its own leaf opening pattern and covers an arc segment of approximately 7°. The available rotation period may be between 15 and 60 s (typically around 20 s). As such, each projection takes between 0.2 and 1 s with all leaves shut for a short time between projections. The delivery assumes constant dose rate of the linac and no dose feedback servo is employed in the current system. The monitor chambers are a safety feature that will terminate irradiation if the dose rate is outside predetermined specifications (typically +/- 5% over 10 s and +/- 50% over 1 s).

The treatment unit also includes a radiation detector system at the beam exit side. This is a Xe-filled ionisation chamber array similar to the ones employed in older diagnostic CT scanners. In practice, it is the tungsten septa that interact most with the MV beam and the secondary electrons generated in the tungsten easily reach the cavities where they are detected. The detector system can be used for acquisition of MVCT scans of the patient in treatment position. The linear accelerator is de-tuned to 3.5 MV and the pulse repetition frequency decreased to keep the dose delivered to the patient during imaging well below 3 cGy. The data acquisition is fast enough to determine the dose given in individual linac pulses and the detector acquisition system (DAS) files are a most useful tool for commissioning and quality assurance (QA) of the unit [36].

### Treatment planning

A treatment file for HT consists of some 60,000 numbers, which specify leaf opening times as a function of gantry position and patient location in the gantry. Due to this complexity, tomotherapy treatment plans can only be created in an inverse planning process. Patient CT data and structure set are transferred to the planning station using DICOM protocol. It is important to extend the planning CT scan at least 5 cm beyond any potential target volume, as the dose delivery may be performed using a 5 cm-wide fan beam. In this case, the ramp up to full dose in the target requires the same length as the fan beam thickness [37]. The outlining tools in the current tomotherapy software are limited to contour modifications but the structures themselves should be created elsewhere. In practice, the number of contours must be typically larger than in ‘conventional’ IMRT, as no beam directions can be pre-determined. The planner chooses positions of the movable red lasers (usually placed on the external marks made during kVCT study), which will be used for initial positioning of the patient on the treatment couch. The planning process allows the specification of multiple targets, which is convenient for simultaneous in-field boost delivery rather than a conventional treatment course given in multiple phases or for the simultaneous treatment of multiple isolated lesions. Treatment delivery and planning depends on parameters specific for HT: fan beam thickness (FBT), pitch factor and modulation factor (MF). The FBT is chosen by the operator to achieve a compromise between fast treatment times and dose modulation in the superior/inferior direction. A large FBT results in larger volumes covered in any projection and a higher central axis dose output while it reduces the scope for conformality and detailed dose modulation in cranio/caudal direction of the patient. As such, the largest FBT of about 50 mm is likely to be used for total body irradiation and mantle type fields while small FBT of 10 mm or even less needs to be employed for small brain lesions [38]. The output in the fan beam drops dramatically below a FBT of 10 mm due to loss of lateral electron equilibrium and partial source occlusion – therefore, it is unlikely that smaller FBTs will be used frequently. A different way to improve the modulation capabilities in the superior/inferior direction is the use of a small pitch factor. The pitch factor is defined as couch movement per rotation in units of the FBT. While it is common to use pitch factors of one or higher in diagnostic CT scanning, the pitch in HT is typically between 0.25 and 0.5 resulting in overlap between adjacent rotations during the helical delivery. The smaller the pitch factor, the longer the treatment; however, a small pitch also improves the capability of dose modulation and the ability to deliver high doses per fraction. A potential problem with large FBT and large pitch is the dose distribution away from the central axis. The beam divergence will cause variations in overlap between adjacent rotations, which increase with distance from the axis of rotation. This is known as the ‘thread effect’. Kissick *et al* have investigated this question and concluded that a pitch factor of 0.86/integer number (e.g., 0.43, 0.287, 0.215, etc.) minimises the thread effect [39].

The MF represents the ratio of maximum leaf opening time to the mean leaf opening time of all MLC leaves, which open in a projection. MF is proportional to the overall treatment time, and with typical physical constraints for the tomotherapy delivery, MFs can be selected between 1 and approximately 6. A small MF results in short treatment times and is adequate for relatively symmetrical targets close to the central axis of the patient, e.g., prostate cancer [40].

**Figure 3:**
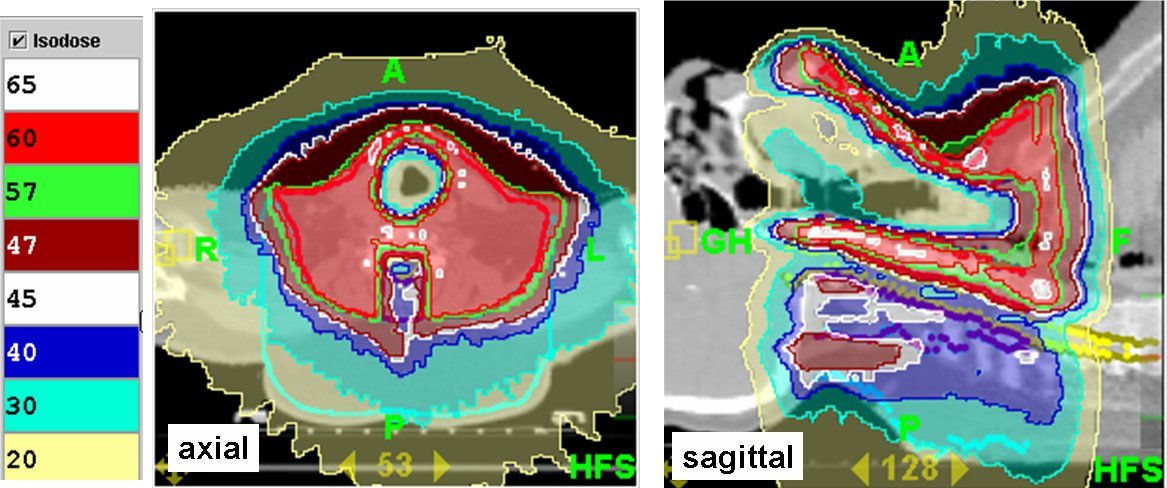
Planned dose distributions in axial and sagittal views of a medullary carcinoma of the thyroid. Note the conformal avoidance of trachea and spinal cord in a patient with microscopic residual disease after resecting medullary carcinoma of the thyroid.

The calculation itself is based on a superposition/convolution dose calculation algorithm [41] and an iterative least square optimisation process [42]. The planning procedure starts with a calculation of the dose distribution produced by all beamlets, which deliver radiation to the target followed by an optimisation of opening times for each leaf guided by precedence, importance and penalty factors. The optimisation results may be quickly modified using the same pre-calculated beamlets and other sets of important and penalty factors. Usually, it takes a couple of hours to produce a plan that would satisfy the requirements of the radiation oncologist. As the tomotherapy environment at present does not allow multitasking, it is generally recommended for performing the dose calculation overnight when multiple calculation tasks can be batched. Figure 3 shows an example of planned dose distribution for an 82-year-old male patient with a resected large medullary carcinoma of the thyroid with microscopic residual disease [planning target volume (PTV) = 1932 cm^3^, target length in sup/inf direction of 13 cm). A dose of 60 Gy to 90% of the PTV was prescribed for delivery in 30 fractions according to the plan where trachea, spinal cord and posterior region were considered sensitive structures with priority to the sparing of spinal cord and trachea.

### MVCT in helical tomotherapy

**Figure 4:**
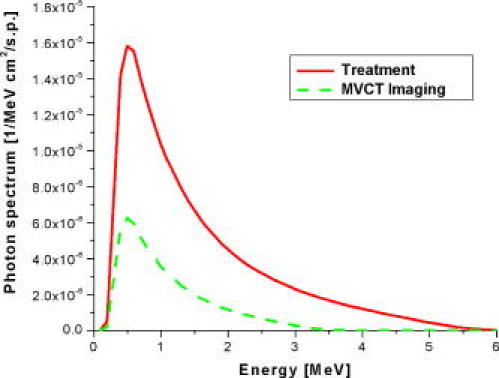
Typical photon beam spectra of helical tomotherapy for two operational modes: treatment mode and MVCT imaging mode. While in the treatment mode, the incident electron energy is approximately 5.7 MeV; in MVCT imaging mode, it is reduced to about 3.5 MeV corresponding to the average photon energies of 1.5 MeV and 1.0 MeV, respectively. Reproduced from [34] with permission.

**Figure 5:**
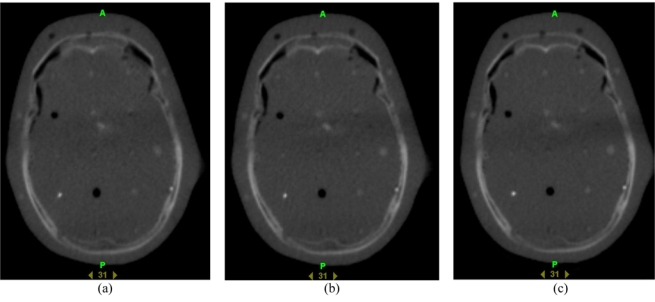
Example of coarse (6 mm interslice distance), normal (4 mm interslice distance) and fine (2 mm interslice distance) options for MVCT imaging of the same slice on a tomotherapy unit.

A patient is initially positioned on the treatment couch using external markings made during the planning kVCT imaging. Then a MVCT is acquired. In the imaging mode, the linear accelerator is detuned in order to improve the soft tissue contrast in such a way that the nominal energy of the incident electron beam is reduced to 3.5 MeV; the resulting photon spectrum is compared in Figure 4 with the spectrum for the treatment mode [34]. This photon beam is collimated by the jaws to a FBT of nominally 4 or 5 mm at the isocenter in superior/inferior direction and 40 cm width laterally. Due to the use of megavoltage X-rays, a further reduction of FBT will result in only a marginal improvement in spatial resolution. Three modes of image acquisition: coarse, normal and fine, obtained by different pitches (couch movement per gantry rotation 12, 8 or 4 mm) are available resulting in image reconstruction with inter-slice distances of 6, 4 and 2 mm. Figure 5 shows MVCT images of a head of Rando phantom taken in coarse (time required to image 18 cm in superior/inferior direction in 30 slices was 156.5 s), normal (time required to image the same volume in 45 slices was 231.5 s) and fine (time required to image a smaller volume in 80 slices was 406.5 s; 80 is the maximum amount of MVCT image slices) imaging options. The image reconstruction matrix for the field of view of 40 cm is 512 (resulting in a 0.78 mm in-plane pixel resolution). The CT detector used in the HT system has been described in several papers [20,43,44]. This arc-shaped xenon detector has 738 channels, each with two ionisation cavities filled with xenon gas and divided by 0.32 mm tungsten septa. The detector array has a 110 cm radius of curvature and 540 out of 738 channels are used for the MVCT image reconstruction. The source to axis distance is 85 cm and the source to detector distance is 145 cm.

**Figure 6:**
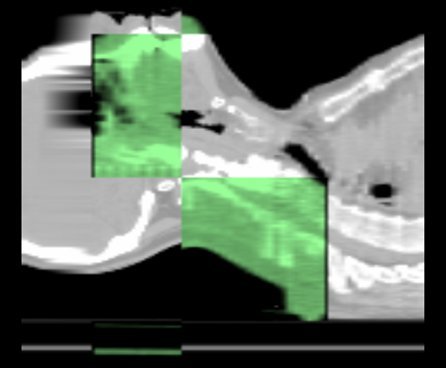
Example of sagittal view of MVCT (green) and kVCT (grey) registration.

Usually the MVCT study is performed using a length, which covers the PTV and/or some specific anatomic landmarks suggested by the physician. Figure 6 shows typical MVCT/kVCT midline sagittal images on an image registration display. The current MVCT images are visually evaluated and registered with the planning kVCT set either automatically or manually. The automatic mode of registration uses a mutual information algorithm. One may choose alignment by translation in three directions and add roll, pitch and yaw displacements as desired. Shifts in superior/inferior and anterior/posterior directions are introduced by couch displacement. Correction in lateral direction is done by the radiation therapists using manual fine adjustment on the treatment couch within the limits of 2.5 cm. Roll correction is accounted by changing the starting angle for gantry rotation [45]. Pitch and yaw corrections can only be introduced by moving the patient and these last two corrections are performed very rarely in clinical practice and only when the other four displacements are not able to provide sufficient alignment. After automatic registration, the alignment of fiducial anatomic features as assigned by a radiation oncologist is checked by the radiation therapists and, if necessary, manual adjustments of the patient setup are performed.

In principle, the field of view (FOV) of 40 cm available in the tomotherapy MVCT system may lead to a degradation of image quality because the tissue outside the FOV is not properly accounted for in the reconstruction process. The typical result is ‘bowl’ artifacts so regarded because the reconstructed CT values are increased in the peripheral regions of the images. Ruchala *et al* have shown that the voxel-based mutual information algorithm used by tomotherapy software for registration still provides successful automatic registration with fields of view down to about one-half of a patient’s size and limited-slice images [46].

Concerning setup uncertainties, it is generally accepted that there are two types, systematic and random. Systematic uncertainties exist because the acquired 3D image may differ from the average target position and random uncertainty is the day-to-day deviation from the target average position [47]. Boswell *et al* compared automatic tomotherapy setup using MVCT to an optically-guided patient positioning system using an anthropomorphic head phantom and found net translational differences between the optical camera and tomotherapy software automatic registration results to be within 2.3 mm in 878 of 900 registration trials [48]. Setup corrections for real patients may be much larger because alignments of organs vary from day to day: the detected maximum setup deviation was 3 mm for patients fixated with the body frame and 6 mm for patients positioned in the vacuum pillow [49].

Performance characteristics of MVCT on Hi-Art tomotherapy system were reported by Meeks *et al* [43]. They studied image noise and uniformity, spatial resolution, contrast properties and multiple scan average dose with a Cardinal Health AAPM CT Performance Phantom (Cardinal Health, Hicksville, NY), which is an acrylic cylinder (21.6 cm in diameter and 31.75 cm in length) with inserts. The images were very uniform with an uniformity index greater than 95% and no statistically significant difference as a function of an equivalent reconstruction matrix or pitch. Typical noise standard deviations are 2-4%, which are only slightly worse than that for diagnostic CT. The visible resolution for the 512 matrix images was approximately 1.25 mm. The contrast resolution e.g., ability to distinguish between muscle tissue with electron density of 3.44-3.48 (1023 electrons/cm^3^ from the surrounding adipose tissue with 3.18 (1023 electrons/cm^3^) is clinically an important characteristic: in general, the need for high resolution is not as pressing as low-contrast detectability [20]. A MVCT scan with the dose of 1.1 cGy allows a clear identification of the prostate and rectum because their electron densities are on the order of 8-10% different from the surrounding region [43]. By increasing the imaging dose, it is possible to improve the contrast e.g., an 8 cGy scan made it possible to delineate regions with the contrast about 2% [20]. This is currently not an option that the user can select in clinical mode. An experimental study comparing MVCT with conventional diagnostic CT scans in dogs with spontaneous tumours concluded that the MVCT image quality is sufficiently good to allow three-dimensional setup verification [29].

### Quality Assurance

**Figure 7:**
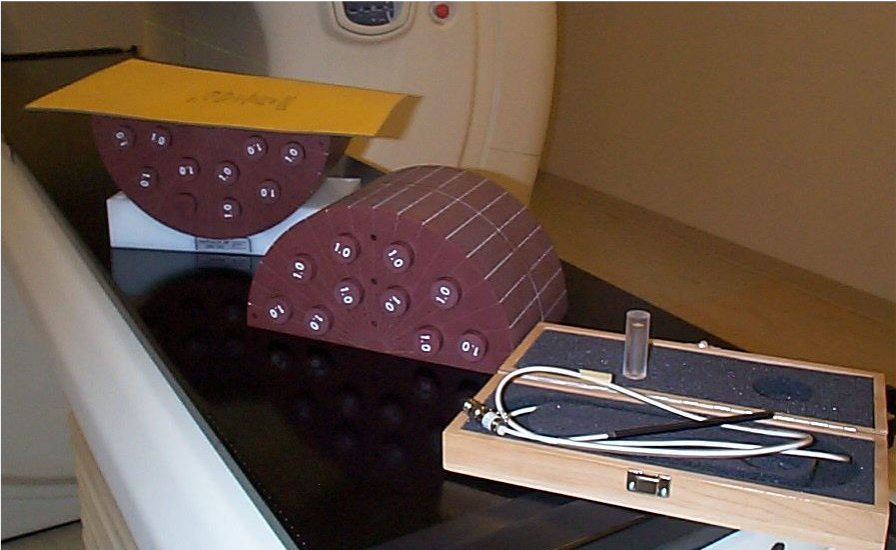
The ‘cheese’ phantom for tomotherapy delivery quality assurance process. Shown is a sheet of EDR2 film (Eastman Kodak Co. Rochester, NY) on the lower half of the phantom. It will be covered with the other half and both half cylinders can be fixed against each other using rubber ties. In the foreground of the photo is the Exradin A1SL ion chamber (Standard Imaging, Middleton, WI), which is used to verify the absolute dose delivered in at least one of the holes drilled in the phantom.

A system of the complexity of a helical tomotherapy unit obviously requires a significant amount of QA. At present, it is left to the user to determine the level of QA as no widely accepted protocol for HT QA exists at present. The suggestion of a QA program for HT is beyond the scope of the present review: see relevant publications [50,51]. The manufacturer acknowledges the need for patient specific QA and it is suggested that the dose distribution for every patient is verified prior to treatment. To this end, a special phantom (‘cheese phantom’ shown in Figure 7) and a QA module in the planning software is included in the purchase of a HT unit. The QA module for planning allows the calculation of the dose distribution, which would be achieved if the patient plan was delivered onto a phantom of the user’s choice. The software is an integral part of the planning station, which makes QA a natural flow of the planning process.

The typical QA process requires the user to verify the absolute dose to at least one point using an ionisation chamber, and the dose distribution in a relevant plane of the phantom using radiographic film. After digitisation, the dose distribution from the film can be directly imported into the planning software and quantitative comparisons can be made with the verification plan using dose profiles and gamma evaluation [52,53].

**Figure 8:**
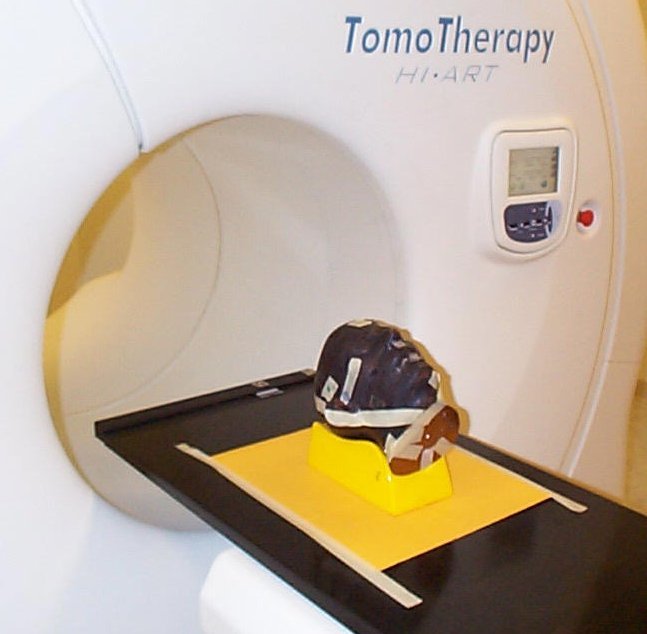
Typical setup of a ‘patient’ (head of Rando phantom) on the top of the film used for *in vivo* dosimetry on tomotherapy unit in London, Ontario, Canada.

**Figure 9:**
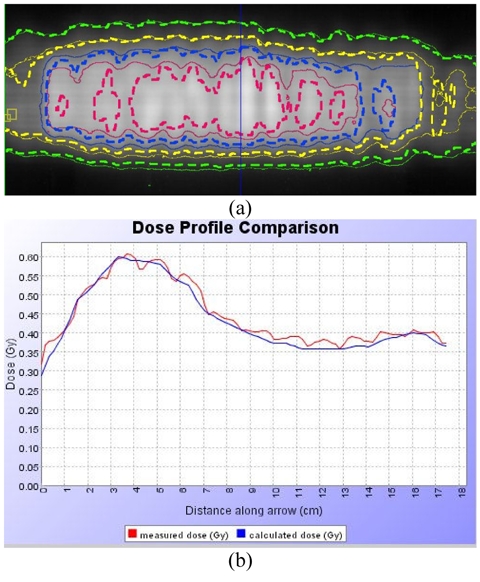
Example of in vivo dosimetry using MVCT study as a ‘phantom’: a) The thin isodose lines represent the dose from the *in vivo* dosimetry film, the thick dotted ones are calculated for the MVCT data on the same day they were imported as a phantom in the DQA software, b) A dose profile comparison along the line shown in (a).

Recently, Kron *et al* have proposed an *in vivo *quality assurance procedure for treatments on the tomotherapy unit [54]. In this method, a film is placed between the patient and the couch top during treatment as can be seen with a phantom example in Figure 8. Tomotherapy Inc. provides a ‘dose delivery quality assurance’ (DQA) module, which re-calculates the dose distribution one would get by delivering the patient treatment sequence onto a selected phantom. It is possible to import MVCT study performed immediately before patient treatment i.e., before the film exposure, as a ‘phantom’. This allows calculation of the dose from the optimised open leaf sinogram for the same patient and utilises the dose comparison tool available in the DQA software as illustrated in Figure 9

In Table 1, we summarize the principle features of helical tomotherapy and compare them with characteristics of conventional radiotherapy units using linear accelerators. In the near future, it is the intent that MVCT will be used also for reconstruction of the dose actually delivered and for planning and re-planning with real-time image collection during treatment delivery [32,55,56].

**Table 1:** Comparison of helical tomotherapy to conventional linac based radiation therapy (RT)

**Feature**	**Conventional Linac based RT**	**Helical Tomotherapy**
Treatment planning	Many commercial systems with different features	Specialised planning system
Treatment options	From single beam to IMRT Electrons and photons	Only IMRT with photons
Beam arrangements	• Different energies possible • Several, typically discrete angles • Two dimensional beams with possible beam modifiers such as wedges or compensators, or IMRT • Non-coplanar arrangements possible	• Only 6 MV photons • 360 degree arc • Fan beam – helical delivery with pitch factor < 1 produces extension of fields in sup/inf direction • Strictly coplanar
MLC and intensity modulation	Shapes the field – multiple segments with different MLC settings and monitor units generate intensity modulated beam	Binary MLC generates beamlet pattern as function of gantry position
Image guidance	Many variations possible – they include kV on board imaging, kV or MV cone beam CT and ultrasound. Most of these systems are add-ons	MVCT using the same radiation source as the treatment unit
Commissioning	Depends on features and options	Partially done in factory – depends on understanding the system
QA	Depends on equipment availability	Integral part of the system

## CONCLUSION

Helical tomotherapy is a new concept in radiation therapy combining IMRT treatment, 3-D inverse treatment planning and 3-D MVCT imaging in one integrated machine. All these components are uniquely designed for IMRT. The complexity of the delivery process only allows inverse treatment planning but delivers highly conformal dose distributions. Treatment planning studies demonstrate dose homogeneity and conformal avoidance capabilities as two of the major strong points of the system. One of the most important features of the HT concept is the on-board MVCT image acquisition system. It allows not only the verification of patient positioning but constitutes a powerful QA tool, which ultimately will yield the reconstruction of the dose as it was actually delivered to the patient on every occasion of a fractionated course of treatment.

## ACKNOWLEDGEMENT

This study was conducted with the support of the Ontario Institute for Cancer Research through funding provided by the government of Ontario.
